# Fermentation, Isolation, Structure, and antidiabetic activity of NFAT-133 produced by *Streptomyces *strain PM0324667

**DOI:** 10.1186/2191-0855-1-42

**Published:** 2011-11-21

**Authors:** Asha A Kulkarni-Almeida, Manoja K Brahma, Prabhu Padmanabhan, Prabhu D Mishra, Rajashri R Parab, Nitin V Gaikwad, Chandni S Thakkar, Pradipta Tokdar, Prafull V Ranadive, Amrutha S Nair, Anagha A Damre, Umakant A Bahirat, Nitin J Deshmukh, Lalit S Doshi, Amol V Dixit, Saji D George, Ram A Vishwakarma, Kumar VS Nemmani, Girish B Mahajan

**Affiliations:** 1 Piramal Life Sciences Limited, 1-Nirlon Complex, Off Western Express Highway, Goregaon (East), Mumbai-400063, Maharashtra State, India; 2 Indian Institute of Integrative Medicine (IIIM), Canal Road, Jammu-180001, Jammu and Kashmir, India

**Keywords:** NFAT-133, *Streptomyces *sp., Antidiabetic, Actinomycetes

## Abstract

Type-2 diabetes is mediated by defects in either insulin secretion or insulin action. In an effort to identify extracts that may stimulate glucose uptake, similar to insulin, a high throughput-screening assay for measuring glucose uptake in skeletal muscle cells was established. During the screening studies to discover novel antidiabetic compounds from microbial resources a *Streptomyces *strain PM0324667 (MTCC 5543, the Strain accession number at Institute of Microbial Technology, Chandigarh, India), an isolate from arid soil was identified which expressed a secondary metabolite that induced glucose uptake in L6 skeletal muscle cells. By employing bioactivity guided fractionation techniques, a tri-substituted simple aromatic compound with anti-diabetic potential was isolated. It was characterized based on MS and 2D NMR spectral data and identified as NFAT-133 which is a known immunosuppressive agent that inhibits NFAT-dependent transcription *in vitro*. Our investigations revealed the antidiabetic potential of NFAT-133. The compound induced glucose uptake in differentiated L6 myotubes with an EC_50 _of 6.3 ± 1.8 μM without activating the peroxisome proliferator-activated receptor-γ. Further, NFAT-133 was also efficacious *in vivo *in diabetic animals and reduced systemic glucose levels. Thus it is a potential lead compound which can be considered for development as a therapeutic for the treatment of type-2 diabetes. We have reported herewith the isolation of the producer microbe, fermentation, purification, *in vitro*, and *in vivo *antidiabetic activity of the compound.

## Background

In 2010, the global prevalence of diabetes was estimated to have reached 285 million and it is predicted to reach 438 million in 2030. Available agents provide imperfect control of the disease, and the medical need for better therapies is widely recognized (Norman 2010). About 90% to 95% of patients have non-insulin dependent diabetes mellitus (NIDDM) or type-2 diabetes and the standard therapy for the treatment of NIDDM has its own limitations (Mark 1997). Diet, oral hypoglycaemic drugs and insulin are the standard modes of treatment. However they are unable to achieve relief from diabetes. This leads to acute and chronic complications. Hence major efforts have been directed towards development of oral hypoglycaemic drugs, to identify both novel insulin secretagogues and compounds able to enhance insulin action in target tissues.

High throughput screening of natural product libraries had previously been attempted by us as an approach towards identifying novel scaffolds with potent biological activity (Kulkarni-Almeida et al. 2008). Several pharmaceutical industries use this approach to investigate natural product based library collections (Quinn et al. 2002). A major segment of these biota collections were represented by microbial cultures. Among the microbial resources actinomycetes have been proven to be a rewarding source of antidiabetic compounds. Acarbose, voglibose, valienamine, adiposin-1, and trestatin-B were reported from *Actinoplanes utahensis *(Schmidit et al. 1977), *Streptomyces hygroscopicus*-*limoneus *(De Melo et al. 2006; Kameda et al. 1980), *S. calvus *(Mahmud 2003; Truscheit et al. 1981), and *S. dimorphogenes *(Yokose et al. 1983; Yokose et al. 1984) respectively. Acarbose is an oral alpha-glucosidase and alpha-amylase inhibitor that was first launched by Bayer in Switzerland in 1989 for the oral treatment of type-2 diabetes mellitus (Schmidit et al. 1977). Voglibose is an alpha-glucosidase inhibitor used for lowering post-prandial blood glucose levels in people with diabetes mellitus. It is produced and marketed in India by trade name Volix^® ^and Vocarb^®^(De Melo et al. 2006). Valielamine, a precursor of voglibose and a new aminocyclitol were isolated from the fermentation broth of *Streptomyces hygroscopicus *subspecies *limoneus*. It has more potent α-glucosidase inhibitory activity against porcine intestinal sucrase, maltase and isomaltase than valienamine, validamine and hydroxyl-validamine which were reported as building blocks of validamycins and microbial oligosaccharide α-glucosidase inhibitors (Kameda et al. 1984; Xu et al. 2009).

One of the hallmarks of type-2 diabetes is decreased sensitivity of cells to insulin. Our approach was to explore microbial collections for extracts with anti-diabetic metabolites that stimulate glucose uptake in skeletal muscle cells. Using this strategy we have previously identified plant extracts *Aegle marmelos *and S*yzygium cumini *which stimulate glucose uptake activity in L6 myotubes. This activity was mediated by activation of glucose uptake by glucose transporter 4 (GLUT4), peroxisome proliferator activator receptor gamma (PPAR-γ) and phosphatidylinositol 3' kinase (PI3K), (Anandharajan et al. 2006). Similarly, (Jung et al. (2006)) showed that *Ganoderma lucidum *extract stimulates glucose uptake activity in skeletal muscles by activating the regulatory molecules PI3-kinase and AMP activated protein kinase (AMPK). We explored the microbial metabolite extract library in search of compounds that enhance glucose uptake in skeletal muscle cells in presence of insulin. Upon insulin treatment of skeletal muscles, the insulin receptor is phosphorylated which activates a signal transduction pathway leading to increased translocation and GLUT4 (Yonemitsu et al. 2001). These features of L6 myotubes are critical since GLUT4 is responsible for insulin-dependent glucose uptake in the mature differentiated skeletal muscle cell.

High throughput screening of diverse microbial metabolite extract library was conducted for detecting glucose uptake inducers in rat skeletal muscle cells. The extract library is prepared from diverse microbes isolated from variety of ecological units in India. 880 extracts representing Actinomycetes strains yielded eight actives. These could stimulate glucose uptake in differentiated skeletal muscle cells. The extract of an actinomycetes strain PM0324667 of the genera *Streptomyces *showed reproducible glucose uptake activity through several refermentation generations. Using fermented broth of this strain, bioactivity guided fractionation was conducted to isolate the active principle. We isolated the compound NFAT-133, which was earlier, reported as an immunosuppressive agent. This compound induced significant glucose uptake in rat skeletal muscle in presence of insulin. The activity was possibly mediated through a PPAR-γ independent mechanism. Further, the compound showed a significant reduction in plasma glucose and plasma insulin levels in diabetic (*db/db*) mice as compared to vehicle treated animals. In this paper, we report the antidiabetic activity of NFAT-133 along with isolation of its producer microorganism, identification, fermentation, and structure elucidation.

## Materials and methods

### Isolation of *Streptomyces *strain PM0324667

The *Streptomyces *strain PM0324667 was isolated from a sandy soil sample collected from Jaipur in Rajasthan, India. The soil was suspended in demineralised water (10% w/v) and heated at 55°C for 6 minutes. 100 μl of the suspension was spread on CSPYME agar consisting of casein (0.1%), corn starch (1.0%), K_2_HPO_4_, (0.05%), peptone (0.1%), yeast extract (0.1%), malt extract (1.0%), agar agar (1.5%), hydroxylamine (0.01%) and amphotericin-B (0.002%) with initial pH value of 7.0. The medium plates were incubated at 30°C for seven days. The colonies were transferred onto agarified ISP2 medium. ISP2 medium consisted of malt extract (1.0%), glucose (0.4%), yeast extract (0.4%) and agar agar (1.6%) with initial pH value of 7.0. The medium slants were incubated at 30°C for 7 days. Typical colonies of streptomycetes, with rough surface and chalky white or gray in color were picked-up on ISP2 agar plates. 47 colonies were isolated and purified from the arid soil sample. All 47 cultures from arid soils were screened in the *in vitro *antidiabetic assay. *Streptomyces *strain PM0324667 was one these 47 which showed consistent and reproducible antidiabetic activity.

### Taxonomy

Microscopic and macroscopic features of the strain PM0324667 were examined by the classical microbiological techniques. Total DNA sample of the strain was prepared as reported (Arun et al. 2005). The 16S ribosomal RNA gene (16S rRNA) was amplified by polymerase chain reaction (PCR) using genomic DNA and universal primers 243F (5'-GGA TGA GCC CGC GGC CTA-3') - forward primer and 1385R (5'-CGG TGT GTA CAA GGC CC-3') - reverse primer and sequenced (Arun et al. 2005). A homology search for the most nearest sequences was performed using the BLAST algorithm on the GenBank/EMBL/DDBJ/PDB.

### Production of NFAT-133 by *Streptomyces *strain PM0324667

*Streptomyces *strain PM0324667 (MTCC 5543) from the ISP2 medium slant was inoculated into seed medium; 274-(1) consisting of glucose (1.5%), corn steep liquor (0.5%), peptone (0.75%), yeast extract (0.75%), calcium carbonate (0.2%) and sodium chloride (0.5%) with initial pH value of 7.0. The culture was incubated on a rotary shaker [240 revolutions per minute (rpm)] at 30°C (± 1°C) for three days. 5 ml of this seed was inoculated into 100 ml fermentation medium; 5189M consisting of malt extract (2.0%), yeast extract (0.2%), glucose (1.0%) and (NH_4_)_2_HPO_4_, (0.005%) with initial pH value of 6.0. The fermentation was carried out on a rotary shaker (240 rpm) at 30°C (± 1°C) for three days.

### Extraction and isolation

The fermented broth was extracted with methanol (1:1, v/v) and it was stirred for 60 minutes and filtered through a coarse filter paper. The filtrate was concentrated using vacuum concentrator. The aqueous concentrate was passed through 10% (v/v) bed volume of Diaion^® ^HP20 (SUPELCO) resin at the rate of 1 L/h. The adsorbent on the resin was eluted with 80% methanol after water wash. The methanolic extract was dissolved in demineralized water to get concentration of 75 mg/ml. 100 ml of this aqueous solution was partitioned with 100 ml ethyl acetate (EA) five times. The EA extracts were pooled together and dried on vacuum concentrator.

The EA concentrate was dry charged to CombiFlash chromatography instrument with pre packed Redisep silica gel column (10 g). The column was sequentially eluted with chloroform and methanol mixture (5-20% methanol) and the fractions monitored by thin layer chromatography (TLC). Similar fractions were pooled to get three samples namely; Fraction-1, Fraction-2 and Fraction-3. These samples along with crude extract of EA were screened for glucose uptake activity. The active Fraction-1 was subjected to reverse phase silica (C-18) cartridge, strata™ from Phenomenex and eluted with water: acetonitrile mixture in gradient mode. The pure compound was recovered from the 20% acetonitrile eluate, by evaporating acetonitrile and water using a high vacuum concentrator.

### Structure elucidation

HPLC was performed using a Lichrosphere RP-18 column (125 × 4 mm) in a Shimadzu LC-2010CHT Liquid Chromatograph. The final purification was done on Waters PrepLC 4000 System. Solvents used for reverse-phase were of HPLC grade and normal-phase column chromatography was performed with demineralized commercial-grade solvents. Silica gel (SiO_2_; 200-300 mesh) was used for CC and GF254 (30-40 mm) TLC plates were procured from Merck. An NMR spectrum of the compound was recorded in CDCl_3 _on Bruker 300 MHz spectrometer with TMS as the internal standard. Chemical shift δ values were expressed in ppm, and coupling constant J in Hz. ESI LC-MS was recorded on Bruker Daltonics. CombiFlash^® ^Sq 16× Teledyne Technologies Company ISCO attached with UV/VIS detector was used for Flash chromatography, using RediSep^® ^Flash Column silica 12 g Teledyne ISCO.

### Large scale fermentation

A well-sporulated seven days old slant culture was inoculated into 200 ml of seed medium consisting of glucose (1.5%), corn steep liquor (0.5%), peptone (0.75%), yeast extract (0.75%), calcium carbonate (0.2%), and sodium chloride (0.5%) in demineralized water with an initial pH value of 6.5-7.0 in 1 L erlenmeyer flasks. All the flasks were incubated on a rotary shaker for 3 days at 230-250 rpm at 30°C (± 1°C). 2.5 L seed was transferred to 150 L fermentor (B. Braun Melsungen AG) containing 100 L production medium consisting of malt extract (2.0%), yeast extract (2.0%), glucose (1.0%), di-ammonium hydrogen phosphate (0.005%) in demineralized water with initial pH value of 6.0 and fermented with the following parameters: aeration 0.5 vvm, temperature 30°C(± 1°C), agitation 100 rpm. 20% dextrose solution was fed at 24 h of the fermentation cycle at 5% (v/v) and the batch was harvested at 48 h. The whole broth was extracted with equal volume of ethyl acetate (EA). The EA extract was used for isolation of the compound NFAT-133.

### Determination of *in vitro *activity

#### Cell lines and Reagents

The cell lines, media and reagents used in the assay are namely; alpha-MEM (Bioconcept), fetal bovine serum (Gibco), penicillin-streptomycin solution (Sigma),^14^C-2-deoxyglucose (GE Healthcare, UK)), 2-deoxyglucose (Sigma Aldrich, St. Louis, MO, USA), KRPH buffer (pH.7.4), Microscint PS (Perkin Elmer, USA), 96-well plates (Nunc), L6 rat skeletal muscle cells (CRL1458, ATCC, USA), human recombinant insulin (Sigma, St. Louis, MO, USA), rosiglitazone (synthesized in house).

#### Assay Procedure

The metabolite extract library samples were screened for the ability to induce glucose uptake in insulin stimulated differentiated L6 myotubes. The L6 rat skeletal muscle cells were seeded into 96 well cell culture plates in complete medium (α-MEM + 10% fetal bovine serum + 1% penicillin-streptomycin solution) and maintained at 5% carbon dioxide (CO_2_) atmosphere. After 48 h, the medium was switched to α-MEM with 2% (v/v) fetal bovine serum (FBS) in order to initiate differentiation. Media change was ensured every 48 h. Compound treatment was conducted once the myoblasts differentiated to form myotubes. The differentiated and fused myotubes were starved in serum free α-MEM for 4 h, treated with samples and further incubated for 24 h. Following treatment and incubation, the cells were sensitized with insulin (200 nM) and incubated for 25 minutes. Radioactive glucose pulsing was done at 0.06 μCi/ml for 10 minutes. The reaction was terminated with cold KRPH buffer. The cell-associated radioactivity was determined by lysing the cells with Microscint PS™ followed by liquid scintillation counting (Huang et al. 2002). The assay was performed in triplicate.

For all experiments, the L6 myoblasts obtained from ATCC were used only up to passage seven and were utilized within two months post revival of a new vial.

#### PPAR-γ luciferase assay

PPAR-γ activation is one of the key mechanisms involved in glucose uptake. To determine if NFAT-133 was exerting its effects by activation of PPAR-γ, a luciferase assay was performed using the expression vector for human PPAR-γ and the PPRE-tk-LUC reporter plasmid. CV1 cells were seeded onto 24 well plates and transient transfections were conducted after over-night incubation using lipofectamine (Invitrogen, CA, USA). Five hours after transfection, the cells were treated with each compound concentration for an additional 24 h, and the luciferase assay (Promega) was performed according to the manufacture's protocol. Luminescence was measured using the Safire2 reader. Transactivation of human PPAR-γ by 1 μM rosiglitazone is considered as 100% activation and hence data for NFAT-133 is calculated in comparison to rosiglitazone which was used as a positive control in the assay. The entire assay was performed in triplicates.

### Determination of *in vivo *activity

#### Animals

Male *db/db *mice (7-9 weeks of age) were housed in individually ventilated cages at room temperature of 22°C ± 2°C, humidity at 55 ± 5%, with a 12:12-h light: dark cycle and throughout the study period had access to water and standard chow *ad libitum*. The guidelines of the Committee for the Purpose of Control and Supervision of Experiments on Animals, Government of India, were followed and the in-house animal ethics committee approved all experimental procedures.

#### Biological Activity

Male *db/db *mice were divided into three groups of 10 animals each with similar body weight and plasma glucose (mean ± S.E.M.) levels. Mice were treated with 0.5% carboxy methyl cellulose (CMC), rosiglitazone (5 mg/kg, p.o.), or NFAT-133 (100 mg/kg, i.p.) twice daily for 10 days. Body weight was measured daily. On day 10, one hour after the last dose, mice were fasted for four hours so as to minimize the variability between the animals. Blood was collected from the fasted mice, plasma was separated by centrifugation (6000 g at 4°C for 7 minutes) and glucose was estimated using a biochemistry auto analyzer (Hitachi Science Systems Limited, Ibaraki, Japan). A small aliquot of separated plasma was stored at -20°C for estimation of insulin using an ELISA assay kit (Linco Research). Immediately after blood sample collection, mice were sacrificed, livers excised and weighed. The data was reproducible in two independent experiments.

#### Statistical analysis

All the results are expressed as Mean ± S.E.M. Statistical analysis was done using GraphPad Prism 4 (version 4.03, GraphPad Software, Inc., CA, USA). For comparison between means of more than two groups one-way analysis of variance (ANOVA) followed by Dunnett's post-hoc analysis was employed.

## Results

### Taxonomy of the *Streptomyces *strain PM0324667

**Figure 1:**
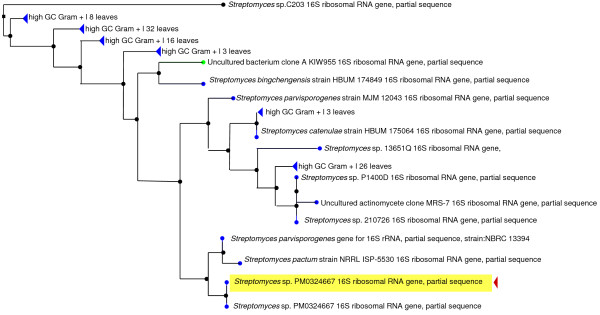
**Phylogeny Tree view for query ID: JF810673, the partial 16S rRNA gene sequence from the of *Streptomyces *strain PM034667**.

The morphological characteristics of the culture *Streptomyces *strain PM0324667 on ISP2 agar were observed. The colonies had a rough surface, marginal areas divided into radial and concentric sections, dry, burst substrate mycelia with profound aerial mycelia, grayish sporulation, convoluted appearance, slightly yellowish diffusible pigment diffusing into the medium to give a buff yellow colour. The partial 16S rRNA gene sequence of the *Streptomyces *strain PM0324667 has been deposited at NCBI and allocated accession # JF810673. This strain showed high similarity in 16S rRNA gene sequence value with *Streptomyces pactum; a*ccession no. EF654095.1 (884/899, 98%). Moreover it showed striking similarity (884/894, 98%) to *Streptomyces parvisporogenes; *accession numbers AB184375.1 and GU350508.1. The full phylogeny tree and blast results are shown in Figure 1 We found that our culture isolate bears closer resemblance to *S. parvisporogenes *as the sequence JF810673 has 98% similarity with the two sequences of S*. parvisporogenes *(Accession numbers: AB184375.1 and GU350508.1). These phenotypic and genotypic properties suggest that the strain PM0324667 belongs to the genus *Streptomyces*. The strain is deposited and preserved at Institute of Microbial Technology, Chandigarh, India (Council of Scientific and Industrial Research, CSIR) under accession number *Streptomyces *sp. MTCC 5543.

### Effect of the ethyl acetate extract of PM0324667 on glucose uptake in differentiated skeletal muscle cells

**Figure 2:**
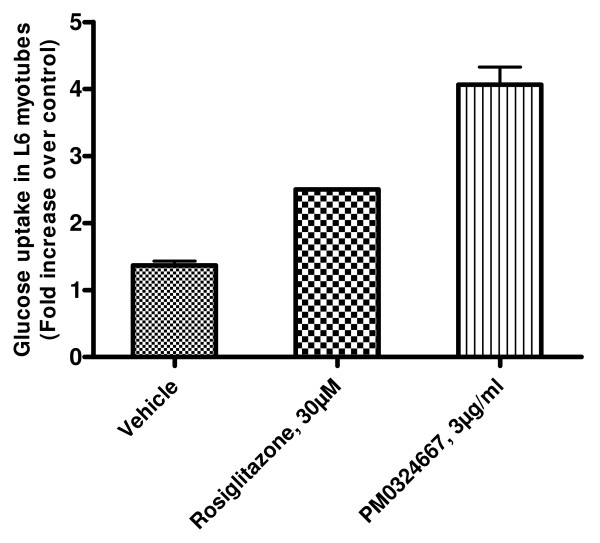
**Effect of the ethyl acetate extract of *Streptomyces *strain PM034667 on glucose uptake in differentiated L6 myotubes sensitized to insulin**.

Insulin resistance in skeletal muscle cells is known to play a pivotal role in the development of diabetes. So we established a high throughput screen using L6 differentiated muscle cells as a model for evaluating the potency of natural product extracts in inducing glucose uptake. High throughput screening of the 20,000 microbial extracts library was conducted. Among the actives the extract from PM0324667 showed a 4-fold increase in glucose uptake activity as compared to control. It induced glucose uptake in differentiated L6 muscle cells in presence of 25 mM glucose and 200 nM insulin. The extract showed consistent and reproducible increase in glucose uptake activity at 3 μg/ml (Figure 2: Where glucose uptake in L6 myotubes exposed to vehicle (0.5% DMSO), glucose uptake in presence of 30 μM rosiglitazone; glucose uptake in presence of 3 μg/ml of the extract of PM034667) when subjected to several refermentation cycles. This effect was comparable to the effect of rosiglitazone at 30 μM (approximately equal to 10.75 μg/ml) in the same experiment.

### Isolation and characterization of NFAT-133

As described in the experimental section, the EA extract of the whole fermentation broth was subjected to bioactivity-guided fractionation to identify the active principle responsible for the glucose uptake activity in PM034667. Three samples based on their fractional similarity were evaluated in the biological assay viz., 1111/38/1, 2 and 3. These samples along with the crude 1111/27 were screened for glucose uptake activity in differentiated skeletal muscle cells. Since bioactivity similar to that of rosiglitazone was observed to be residing in a single fraction 1111/38/1, the same fraction was taken up for further isolation of the active principle (Table 1).

**Table 1:** Effect of the ethyl acetate extract (111/27/E.A) of *Streptomyces *strain PM034667 and fractions 1111/38/1, 2 and 3 on glucose uptake in differentiated L6 myotubes

Sample #	Glucose uptake in L6 myotubes* (Fold increase over control at 3 μg/ml)	Inference
EA- crude	2.5	Active
Fraction-1	2.4	Active
Fraction-2	1.4	Not active
Fraction-3	1.5	Not active
Rosiglitazone (4 μg/ml)	1.8	Active

*: n = 3

NFAT-133 was isolated by fermentation of the *Streptomyces *culture PM0324667 using optimized fermentation media and conditions as described in the experimental section. The biological activity coincided with the profile on a LC-ESI positive ion mass spectrum which showed a distinct peak m/z at 299 [M + Na]^+ ^suggesting a molecular weight of the compound was 276 (Additional file 1). The compound was a pale yellow viscous oil with a UV absorption maxima of 212 nm, and molecular formula; C_17_H_24_O_3_.

A^13^C NMR spectra (Table 2, Additional file 2) indicated eight carbons in the range of δ 128-140, these signals were characteristic of C = C system present in the compounds. Another carbon signal at δ 214 indicated a ketone unit in the structure. Two carbons at δ 63.8 and 74.6 were assigned as CH-OH and CH_2_-OH with the help of HSQC (Additional file 3) and DEPT-135 spectra (Additional file 4) of the compound NFAT-133.

**Table 2:** NMR assignment for NFAT-133 in CDCl_3_

Carbon#	^13^C NMR	^1^H NMR	Reported^13^C NMR	Reported^1^H NMR
1	^63.8 (CH^2^)^	4.3, bs	^63.7(CH^2^)^	4.2, dd
2	131.6 (CH)	6.2, dt	132.8(CH)	6.1, dt
3	128.9 (CH)	6.9, d	129.3(CH)	6.9 dt
4	136.1 (Q)	--	137.2(Q)	--
5	128.0 (CH)	7.2, Overlap	128.6(CH)	7.2, d
6	135.9 (Q)	--	137.0(Q)	--
7	128.7(CH)	7.07, dd	129.7(CH)	7.07 dd
8	126.6 (CH)	7.05, dd	128.0(CH)	7.05, d
9	138.6 (Q)	--	140.2 (Q)	--
10	37.3 (CH)	3.1, m	39.8(CH)	3.08, dq
11	74.6 (CH)	4.1, m	76.6(CH)	4.2, dd
12	48.5(CH)	2.5, m	51.2(CH)	2.3, qd
13	214 (Q)	--	214.2 (Q)	--
14	^29.3 (CH^3^)^	2.1, s	^28.4(CH^3^)^	2.05, s
15	^21.1(CH^3^)^	2.3, s	^21.0 (CH^3^)^	2.2, s
16	^17.5(CH^3^)^	1.3, d	^19.2(CH^3^)^	1.2, d
17	^10.8(CH^3^)^	1.09, d	^9.7(CH^3^)^	0.9, d

The NMR data for NFAT-133

**^1^H NMR (500 MHz, CDCl_3_)**: δ 7.26 (1H, overlap, H-5), 7.07 (1H, d, H-7), 7.05 (1H, d, H-8), 6.9 (1H, d, J = 15.5 Hz, H-3), 6.2 (1H, dt, J = 15.5 Hz, H-2), 4.37 (2H, bs, H-1), 4.11 (1H, bs, H-11), 3.12 (1H, m, H-10), 2.52 (1H, m, H-12), 2.35 (3H, s, H-15), 2.14 (3H, s, H-14), 1.34 (3H, d, J = 7.0 Hz, H-16), 1.09 (3H, d, J = 7.0 Hz, H-17),

**^13^C NMR (75 MHz, CDCl_3_)**: δ 214 (C = O), 138.6 (C, C-9), 136.13 (C, C-4), 135.9 (C, C-6), 131.6 (CH, C-2), 128.9 (CH, C-3), 128.74 (CH, C-7), 128.0 (CH, C-5), 126.6 (CH, C-8), 74.65 (CH, C-11), 63.83 (CH_2_, C-1), 48.57 (CH, C-12), 37.3 (CH, C-10), 29.3 (CH_3_, C-14), 21.11 (CH_3_, C-15), 17.58 (CH_3_, C-16), 10.8 (CH_3_, C-17).

The^1^H NMR (Additional file 5) and COSY spectra suggested the presence of trisubstituted benzene ring with coupled proton signal at δ 7.2 (C-5), 7.07 (C-7) and 7.05 (C-8). A proton signal at δ2.3 singlets was assigned as Me group at C-6 position. The olefinic protons at C-3, C-4 and the only CH_2 _unit at C-1 were well correlated in COSY spectrum.

**Figure 3:**
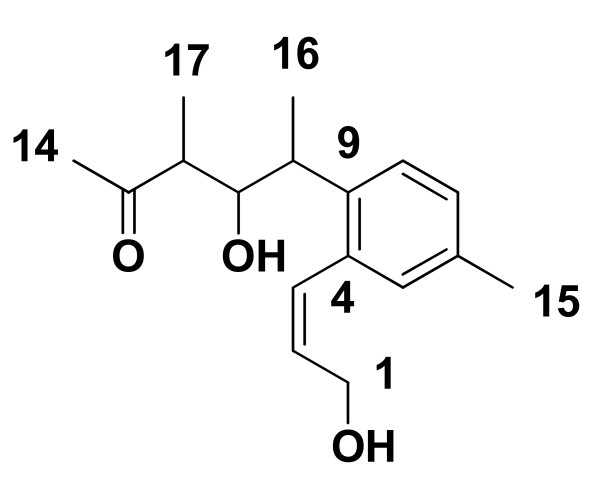
**Structure of NFAT-133**.

The other proton signals were also assigned to their respective carbons with the help of HSQC, COSY and DEPT spectra of the compound. The complete interpretation of data was in full agreement with the reported values of the compound NFAT-133 (Figure 3) (Burres et al. 1995; Qureshi et al. 2001).

### Effect of NFAT-133 treatment *in vitro*

**Figure 4:**
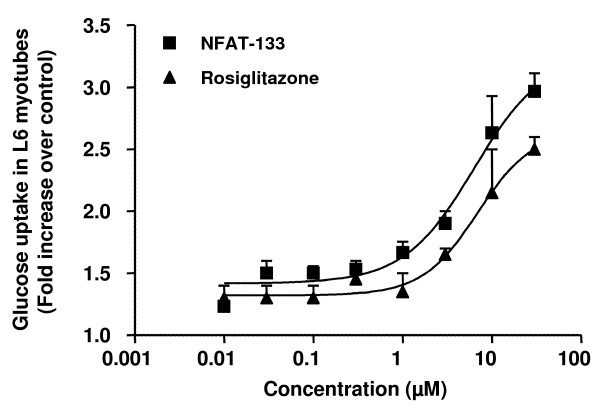
**Dose dependent increase in glucose uptake activity in differentiated L6 mytubes sensitized with insulin and exposed to log dose concentrations of NFAT-133 (-■-) and rosiglitazone (-▲-)**.

Bioactivity guided isolation of the active compounds resulted in isolation of the compound NFAT-133. This compound has been previously isolated from a *Streptomyces *strain AB 2184C-502 and derived its name NFAT-133 due to its ability to inhibit the activation of the transcription factor - NFAT in Jurkat T-cells and block proliferation of splenocytes derived from Balb/c and C57BL/6 mice *in vitro *(Burres et al.1995). Herewith we report its effect on glucose uptake in differentiated skeletal muscle cells. NFAT-133 induces increased glucose uptake in L6 myotubes in presence of insulin (EC_50 _= 6.3 ± 1.8 μM). This effect is comparable to the effect of rosiglitazone (EC_50 _= 6.9 ± 1.8 μM) under similar experimental conditions (Figure 4). However, unlike rosiglitazone, NFAT-133 does not significantly affect PPAR-γ activity (Table 3). Rosiglitazone is a full agonist of PPAR-γ at 1 μM. Whereas NFAT-133 even at concentrations as high as 100 μM, does not induce PPAR-γ activity, indicating that NFAT-133 stimulates glucose uptake via mechanisms distinct from that of rosiglitazone.

**Table 3:** Effect of NFAT-133 on PPAR-γ activity in human PPAR-γ receptor transfected CV1 cells

Compound	% hPPAR-γ activation
NFAT-133, 100 μM*	9.0
NFAT-133, 30 μM*	0.2
Rosiglitazone, 1 μM*	100

*: n = 3

### Effect of NFAT-133 treatment *in vivo*

**Figure 5:**
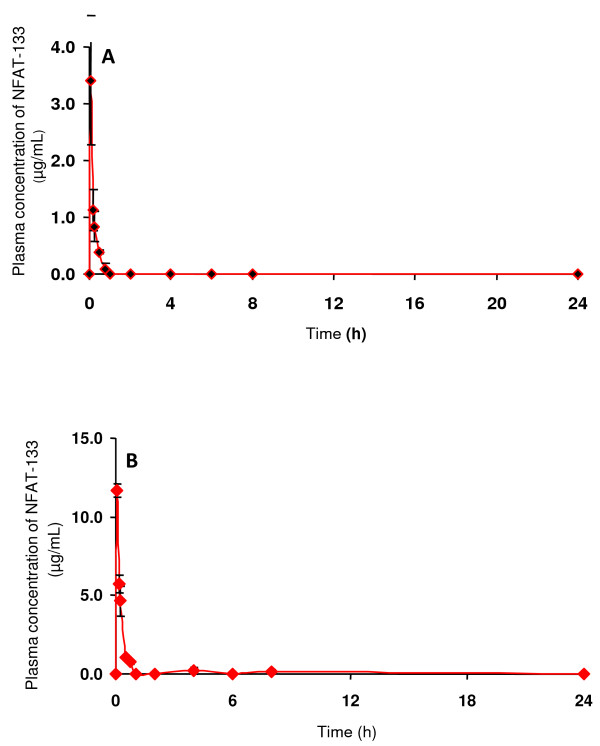
**Pharmacokinetic data of NFAT-133 by oral (A) and intraperitoneal (B) absorption in female *db/db *mice at 30 mg/kg**.

Rosiglitazone treatment induces glucose uptake in insulin-resistant rat skeletal muscle cells by activating PPAR-γ and by increasing the resident AMPKα2 activity of these cells. Similarly, troglitazone a PPAR-γ dual agonist increases glucose uptake by inducing GLUT4 translocation in L6 skeletal muscles (Lessard et al. 2006; Yonemitsu et al. 2001). However, these compounds are PPAR-γ agonists, and have several side-effects that cause increased body weight and hepaptoxicity on chronic exposure. Since NFAT-133 increases glucose uptake in L6 myotubes and does not agonise PPAR-γ receptors significantly, we decided to investigate its *in vivo *profile in diabetic animals. Prior to planning an efficacy study we determined the pharmacokinetic profile of NFAT-133 (Figure 5, Table 4) using female *db/db *mice with n = 4 per time point. NFAT-133 was found to have poor oral bioavailability. But, since the compound appeared to have a novel mechanism of action, we decided to explore its *in vivo *efficacy potential by attempting the intraperitoneal route of administration. NFAT-133 administered by the intraperitoneal route achieved plasma concentrations higher than the *in vitro *EC_50 _in skeletal muscles. The dose selected, was 20 times higher than that of rosiglitazone on the basis of the pharmacokinetic profile of the compound, so as to have the sufficient exposure to show the pharmacological effect.

**Table 4:** Pharmacokinetic (PK) profile of NFAT-133 (30 mg/kg) administered orally and intraperitoneally in female *db/db *mice

PK	Oral	IP
T max (h)	0.08	0.08

C max (μg/ml)	3.41	11.653

C max (μM)	12.34	42.221

AUC last	0.63	3.217

AUC 0-infinity	0.65	3.553

T1/2 (h)	0.16	1.86

IP: intraperitoneal, T_max _(h): Time to maximum observed plasma concentration, C_max_: Maximum observed plasma concentration, AUC_last: _Area under the concentration-time curve from time 0 to the time of last observed concentration, AUC_0-infinity_: Area under the concentration-time curve from time 0 to infinity, and T_1/2_: Terminal half-life.

**Figure 6:**
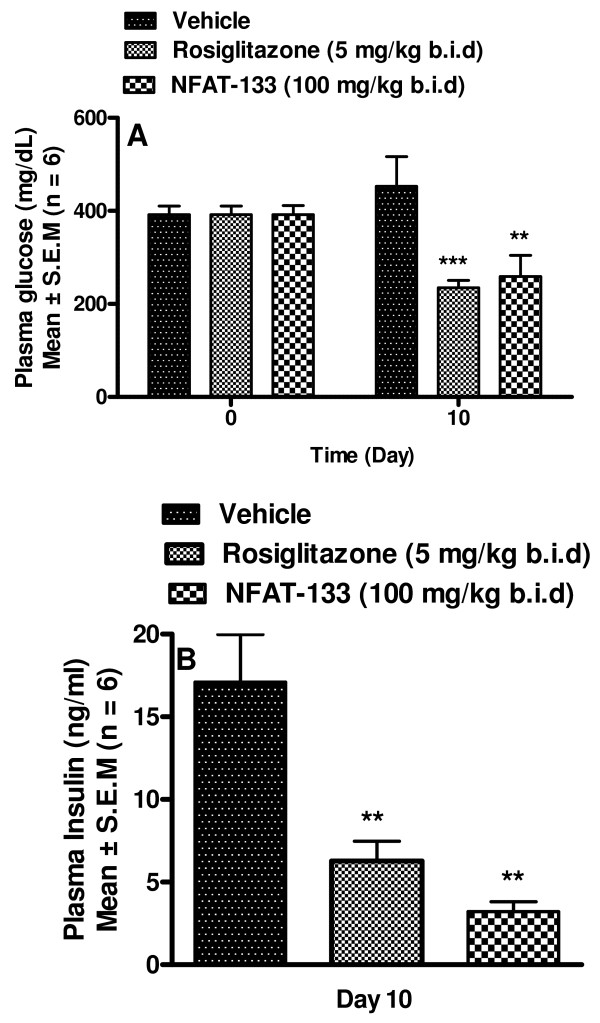
**Effect of NFAT-133 on plasma glucose and insulin in *db/db *mice**. *db/db *mice were treated for 10 days, bis in die (b.i.d) with vehicle, NFAT-133 [100 mg/kg, intra-peritoneal (i.p)] or rosiglitazone [5 mg/kg, per os (p.o.)]. Plasma glucose (A) and insulin (B) were measured after 10 days of treatment. All the results are expressed as mean ± standard error of the mean (S.E.M). *** P value < 0.001 vs. vehicle group.

NFAT-133 showed a significant reduction in plasma glucose and plasma insulin levels as compared to vehicle treated animals. The decrease in plasma glucose and insulin in the NFAT-133 group was similar to that observed in rosiglitazone treated group (Figure 6). The liver weight of rosiglitazone treated animals was significantly increased as compared to vehicle treated group, (Table 5) (2.3 ± 0.1 g vs. 2.7 ± 0.1 g), while NFAT-133 had no effect on liver weight (2.0 ± 0.1 g). In addition, rosiglitazone showed significant increase in body weight, which is the well known adverse effect of PPAR-γ agonists. Our compound NFAT-133 showed significant reduction in body weight gain as compared to the vehicle treated animals (% change in body wt: 6.02 ± 0.64, 14.63 ± 0.85 and -1.08 ± 1.21 in vehicle, rosiglitazone and NFAT-133, respectively). This is a beneficial effect over the compounds belonging to the class of PPAR-γ agonists; e.g., rosiglitazone (Table 5).

**Table 5:** Effect of NFAT-133 and rosiglitazone on the body weight and liver weight in response to treatment in *db/db *mice

Group	% Change in body weight	% Change in liver weight
Vehicle	6.0 ± 0.6	2.3 ± 0.1

Rosiglitazone, 5 mg/kg, po	14.6 ± 0.8**	2.6 ± 0.1*

NFAT-133, 100 mg/kg, ip	-1.1 ± 1.2**	2.0 ± 0.1*

Mice were treated for 10 days, bis in die (b.i.d) with vehicle, NFAT-133 [100 mg/kg, intra-peritoneal (i.p)] or rosiglitazone [5 mg/kg, per os (p.o.)]. The percent change in liver and bidy weight were measured after 10 days of treatment. All the results were expressed as Mean ± S.E.M. (n = 10). ** p < 0.01 and * p < 0.05 *vs*. day 0.

## Discussion

*Streptomyces pactum *was known to produce antitumor compounds like pactamycin (Bhuyan 1962), vasodilating secondary metabolite like actinopyrones A, B and C (Yano et al. 1986), NADH oxidase inhibitors such as piericidin class of compounds (Matsumoto et al. 1987), and polyetheric antibiotics such as lonomycin (Hamill et al. 1989). Similarly *Streptomyces parvisporogenes *has been reported to synthesize polyenic antifungal agent like Antibiotic PA 616 (Chas 1960) and pepstatin class of compounds which are pepsin inhibitors (Umezawa 1970). There has been no report of NFAT-133 or similar class of compounds isolated from strains of *Streptomyces pactum *or *Streptomyces parvisporogenes *to date.

Our screening program for new compounds has yielded the compound NFAT-133. NFAT-133 elicits a significant and dose dependent glucose uptake response in differentiated L6 cells. In skeletal muscles the glucose uptake is mediated by activation of the transporter molecule GLUT4 (Wijesekara et al. 2006). GLUT4 activation is controlled by regulatory proteins which activate either the AMPK or PI3K pathway. Several natural products have been earlier reported to elicit similar responses in L6 skeletal muscle cells. We have shown that the plant extracts *Aegle marmelos *and *Syzygium cumini *stimulate glucose uptake in skeletal muscle by augmenting the activity of the regulatory molecules; phosphatidyl inositol-3-kinase (PI3K), AMPK and GLUT-4 (Anandharajan et al. 2006). *Ganoderma lucidum *extract stimulates glucose uptake in L6 rat skeletal muscle cells by stimulating the activity of PI3K as well as a PI3K independent pathway which is controlled by the AMP activated protein kinase (Kamei et al. 2002; Jung et al. 2006; Cheng et al. 2006). It is possible that NFAT-133 may be mediating its effects through some of these pathways since the compound did not significantly activate PPAR-γ. PPAR-γ is a major signaling molecule that is implicated in glucose uptake by skeletal muscle cells *in vivo*. However, activation of this receptor has several drawbacks. This has been noted especially in the case of thiazolidinediones which are very successful in reducing the clinical symptoms of diabetes. However, their major drawback is that they mediate their effects through activation of the PPAR-γ family of nuclear receptors. The most significant side effects of this group of compounds include weight gain, hepatotoxicity and cardiovascular manifestations. The fact that our compound NFAT-133 did not induce glucose uptake through activation of PPAR-γ is significant. Hence, we evaluated the efficacy of the molecule in reducing systemic blood glucose levels in *db/db *mice. Our data showed that NFAT-133 reduces systemic blood glucose levels. This was correlated with a simultaneous decrease in plasma insulin. Though actinomycetes produce few but prominent antidiabetic compounds, our report of NFAT-133 activity has revealed that further exploration of actinomycetes group will provide an avenue for discoveries leading to new medicines for diabetes. In conclusion, we found that NFAT-133, a natural product isolated from Streptomycetes, stimulates glucose uptake in L6 myotubes. This mechanism might explain its antihyperglycemic effects in diabetic animals.

## Conclusions

Antihyperglycemic effects of the Streptomycetes compound NFAT-133 has been revealed by *in vitro *and animal studies.

## Competing interests

The authors declare that they have no competing interests.

## Authors' contributions

Microbiology and fermentation: GBM, SDG, RRP, PVR, PT, *In vitro *screening and analysis: AAK, MKB, PP, CST, ASN, *In vivo *evaluation: KVN, AAD, UAB, NJD, LSD, AVD, Chemical isolation and structural elucidation: PDM, NVG, RAV

## Supplementary Material

Additional file 1 **ESI-MS of the compound NFAT-133**. The chromatogram represents the molecular mass of the isolated compound NFAT-133 from the *Streptomyces *strain PM0324667. The sample ID for the compound was: 1111- 41-1. Click here for file

Additional file 2 **^13^C NMR of the compound NFAT-133**. The chromatogram represents the^13^C NMR of the isolated compound NFAT-133 from the *Streptomyces *strain PM0324667. The sample ID for the compound was: 1111-41-1. Click here for file

Additional file 3 **^13^C HSQC of the compound NFAT-133**. The chromatogram represents the^13^C HSQC of the isolated compound NFAT-133 from the *Streptomyces *strain PM0324667. The sample ID for the compound was: 1111-41-1. Click here for file

Additional file 4 **DEPT-135 of the compound NFAT-133**. The chromatogram represents the DEPT-135 of the isolated compound NFAT-133 from the *Streptomyces *strain PM0324667. The sample ID for the compound was: 1111-41-1. Click here for file

Additional file 5 **^1^H NMR of the compound NFAT-133**. The chromatogram represents the^1^H NMR of the isolated compound NFAT-133 from the *Streptomyces *strain PM0324667. The sample ID for the compound was: 1111-41-1. Click here for file

## References

[B1] Anandharajan R Jaiganesh S Shankernarayanan NP Viswakarma RA Balakrishnan A In vitro glucose uptake activity of *Aegeles marmelos *and Syzygium cumini by activation of Glut-4, PI3 kinase and PPAR-γ in L6 myotubes Phytomedicine 2006 13 434 441 10.1016/j.phymed.2005.03.008 16716914

[B2] Arun DS Jaspreet S A nonenzymatic method to isolate genomic DNA from bacteria and actinomycete Analytical Biochemistry 2005 337 2 354 356 10.1016/j.ab.2004.11.029 15691519

[B3] Bhuyan BK Pactamycin Production by *Streptomyces pactum* Appl Microbiol 1962 10 4 302 304 13868850 10.1128/am.10.4.302-304.1962 PMC1057863

[B4] Burres NS Premachandran U Hoselton S Cwik D Hochlowski JE Ye Q Sunga GN Karwowski JP Jackson M Whittern DN Mcalpine JB Simple aromatics identified with a NFAT-lacZ transcription assay for the detection of immunosuppressants J Antibiot 1995 48 5 380 386 7797439 10.7164/antibiotics.48.380

[B5] Chas. Pfizer and Co Antibiotic designated compound 616 from *streptomyces parvisporogenes* Inc. British Patent 832391 1960

[B6] Cheng Z Pang T Gu M Gao AH Xie CM Li JH Nan FHL Berberine-stimulated glucose uptake in L6 myotubes involves both AMPK and p38 MAPK Biochim Biophys Acta 2006 1760 11 1682 1689 10.1016/j.bbagen.2006.09.007 17049164

[B7] De Melo EB Gomes ADS Carvalho I α- and β-Glucosidase inhibitors: chemical structure and biological activity Tetrahedron 2006 62 44 10277 10302 10.1016/j.tet.2006.08.055

[B8] Hamill RL Yao RC Process for producing antibiotic A80438 U.S. patent 4,830,967 1989

[B9] Huang C Somwar R Patel N Niu W Torok D Klip A Sustained Exposure of L6 Myotubes to High Glucose and Insulin Decreases Insulin-Stimulated GLUT4 Translocation but Upregulates GLUT4 Activity Diabetes 2002 51 2090 2098 10.2337/diabetes.51.7.2090 12086937

[B10] Jung KH Ha E Kim MJ Uhm YK Kim HK Hong SJ Chung JH Yim SV Ganoderma lucidum extract stimulates glucose uptake in L6 rat skeletal muscle cells Acta Biochimica Polonica 2006 53 3 597 601 16964326

[B11] Kameda Y Asano N Yoshikawa M Matsui K Valienamine as an α-glucosidase inhibitor J Antibiot 1980 33 12 1575 1576 6788743 10.7164/antibiotics.33.1575

[B12] Kameda Y Asano N Yoshikawa M Takeuchi M Yamaguchi T Matsui K Horii S Fukase H Vaholamine, a new α-glucosidase inhibiting aminocyclitol. produced by *Streptomyces hygroscopicus* J Antibiot 1984 7 11 1301 1307 10.7164/antibiotics.37.1301 6392268

[B13] Kamei R Kitagawa Y Kadokura M Hattori F Hazeki O Ebina Y Nishihara T Oikawa S Shikonin Stimulates Glucose Uptake in 3T3-L1 Adipocytes via an Insulin-Independent Tyrosine Kinase Pathway Biochem Biophys Res Commun 2002 292 642 651 10.1006/bbrc.2002.6714 11922615

[B14] Kulkarni-Almeida AA Suthar A Goswami H Vishwakarma R Chauhan VS Balakrishnan A Sharma S Novel leads from Heliotropium ovalifolium, 4,7,8-trimethoxy-naphthalene-2-carboxylic acidand6-hydroxy-5,7-dimethoxy-naphthalene-2-carbaldehyde showspecific IL-6inhibitoryactivityinTHP-1 cells and primary human monocytes Phytomedicine 2008 15 1079 1086 10.1016/j.phymed.2008.04.013 18583119

[B15] Lessard SJ Chen ZP Watt MJ Hashem M Reid JJ Febbraio MA Kemp BE Hawley JA Chronic rosiglitazone treatment restores AMPK_2 activity in insulin-resistant rat skeletal muscle Am J Physiol Endocrinol Metab 2006 290 E251 E257 16118254 10.1152/ajpendo.00096.2005

[B16] Mahmud T The C_7_N aminocyclitol family of natural products Nat Prod Rep 2003 20 1 137 166 10.1039/b205561a 12636088

[B17] Mark M Grell W Hypoglycaemic effects of the novel antidiabetic agent repaglinide in rats and dogs Br J Pharmacol 1997 121 1597 1604 10.1038/sj.bjp.0701307 9283692 PMC1564864

[B18] Matsumoto M Mogi K Nagaoka K Ishizeki S Kawahara RN New piericidin glucosides, glucopiericidins A and B J Antibiot (Tokyo) 1987 40 2 149 56 10.7164/antibiotics.40.149 3570963

[B19] Norman P Diabetes Pipeline: Intense Activity to Meet Unmet Need Report- Overview 2010 Cambridge Healthtech Institute, Massachusetts (USA)

[B20] Quinn RJ Almeida LPDe Guymer G Hooper JNA Australian biodiversity via its plants and marine organisms - A high-throughput screening approach to drug discovery Pure Applied Chemistry 2002 74 4 519 526 10.1351/pac200274040519

[B21] Qureshi A Mauger JB Cano RJ Galazzo JL Lee MD MF-EA-705a and MF - EA - 705b, new metabolites from microbial fermentation of Streptomyces sp J Antibiot 2001 54 12 1100 1103 11858667 10.7164/antibiotics.54.1100

[B22] Schmidit DD Frommer W Junge B Muller L Wingender W Truscheit E Schafer D α-Glucosidase inhibitors Naturwissenschaften 1977 64 10 535 536 10.1007/BF00483561 337162

[B23] Truscheit E Frommer W Junge B Muller L Schmidt DD Wingender W Chemistry and Biochemistry of Microbial -Glucosidase Inhibitors Angew Chem Int Ed Engl 1981 20 744 761 10.1002/anie.198107441

[B24] Umezawa H Aoyagi T Morishima H Matsuzaki M Hamada M Takeuchi T Pepstatin, a new pepsin inhibitor produced by agtinomygetes J Antibiot (Tokyo) 1970 23 259 262 10.7164/antibiotics.23.259 4912600

[B25] Wijesekara N Thong FSL Antonescu CN Klip A Diverse Signals Regulate Glucose Uptake into Skeletal Muscle Can J Diabetes 2006 30 1 80 88

[B26] Xu H Yang J Bai L Deng Z Mahmud T Genetically engineered production of 1,1'-bis-valienamine and validienamycin in *Streptomyces hygroscopicus *and their conversion to valienamine Appl Microbiol Biotechnol 2009 81 5 895 902 10.1007/s00253-008-1711-z 18820907 PMC2610235

[B27] Yano K Yokoi K Sato J Oono J Kouda T Ogawa Y Nakashima T Pactamycin Production by *Streptomyces pactum* J Antibiot 1986 34 1 32 37 10.7164/antibiotics.39.32 3753969

[B28] Yokose K Ogawa K Sano T Watanabe K Maruyama HB Suhara Y New α-amylase inhibitor, Trestatins I. Isolation, characterization and biological activities of Trestatins A, B and C J Antibiot 1983 36 9 1157 1165 6605333 10.7164/antibiotics.36.1157

[B29] Yokose K Ogawa M Ogawa K New α-amylase inhibitor, Trestatins III. Structure determination of New Trestatin Components Ro 09-0766, Ro 09-0767 and Ro 09-0768 J Antibiot 1984 37 2 182 186 6608512 10.7164/antibiotics.37.182

[B30] Yonemitsu S Nishimura H Shintani M Inoue R Yamamoto Y Masuzaki H Ogawa Y Hosoda K Inoue G Hayashi T Nakao K Troglitazone Induces GLUT4 Translocation in L6 Myotubes Diabetes 2001 50 1093 1101 10.2337/diabetes.50.5.1093 11334413

