# Supplementary material for: Fermentation, Isolation, Structure, and antidiabetic activity of NFAT-133 produced by Streptomyces strain PM0324667
Source: AMB Express. 2011 Nov 21;1:42. doi: 10.1186/2191-0855-1-42 (PMC3274447; doi:10.1186/2191-0855-1-42)

Analysis Info

Instrument : ID : AS - I -08

Method	
Analysis Name	40333.m
Sample Name	1111-41-1
Comment	

**Acquisition Date**	6/24/2008 9:46:07 AM
**Operator**	Prajakta
**Instrument**	esquire4000

Mode

Mass Range Mode	Std/Normal
Ion Polarity	Positive
Ion Source Type	ESI
Alternating Ion Polarity	off
Current Alternating Ion Pol	Positive
Divert Valve	to Source

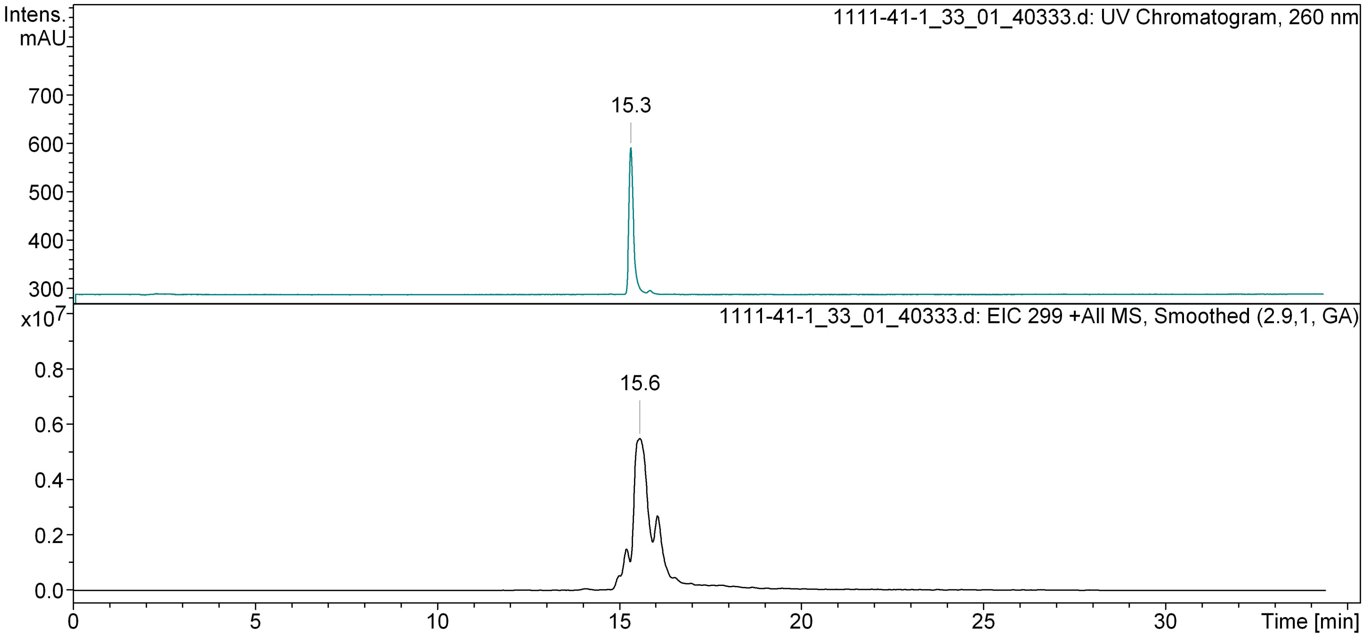

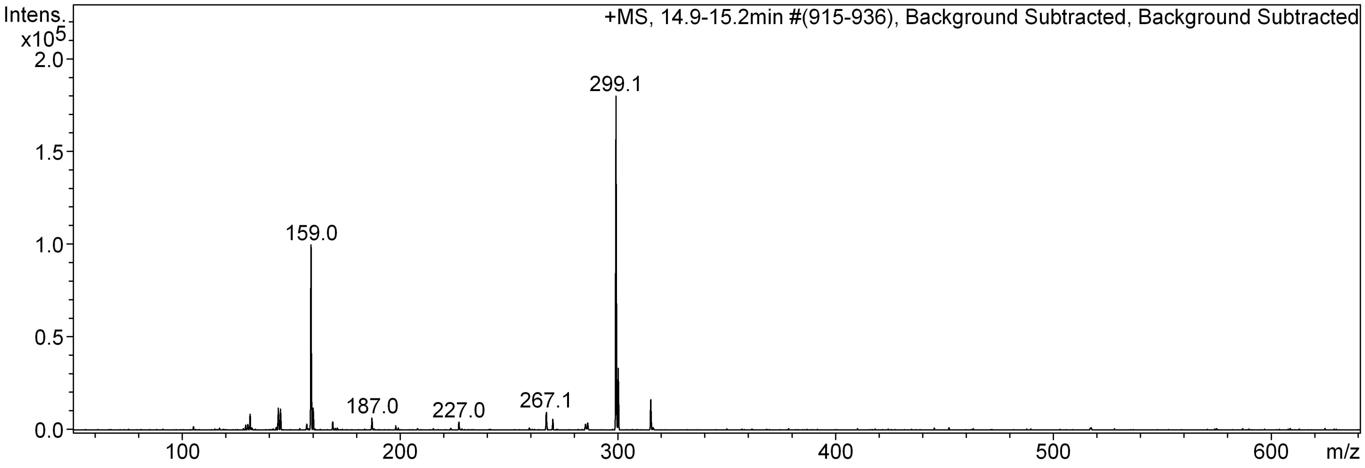

Supplement: Additional file 1 — ESI-MS of the compound NFAT-133. The chromatogram represents the molecular mass of the isolated compound NFAT-133 from the Streptomyces strain PM0324667. The sample ID for the compound was: 1111- 41-1. [file 2191-0855-1-42-S1.PDF]
