# Supplementary material for: Fermentation, Isolation, Structure, and antidiabetic activity of NFAT-133 produced by Streptomyces strain PM0324667
Source: AMB Express. 2011 Nov 21;1:42. doi: 10.1186/2191-0855-1-42 (PMC3274447; doi:10.1186/2191-0855-1-42)
Supplement: Additional file 2 — 13C NMR of the compound NFAT-133. The chromatogram represents the13C NMR of the isolated compound NFAT-133 from the Streptomyces strain PM0324667. The sample ID for the compound was: 1111-41-1. [file 2191-0855-1-42-S2.PDF]

Piramal Life Sciences Limited

Sample : 1111-41-1
Solvent : CDCl3
Spectrum : 13C Spectrum
Instrument: AS-I-10
Analyst : Jingal
Date : 26.06.2008

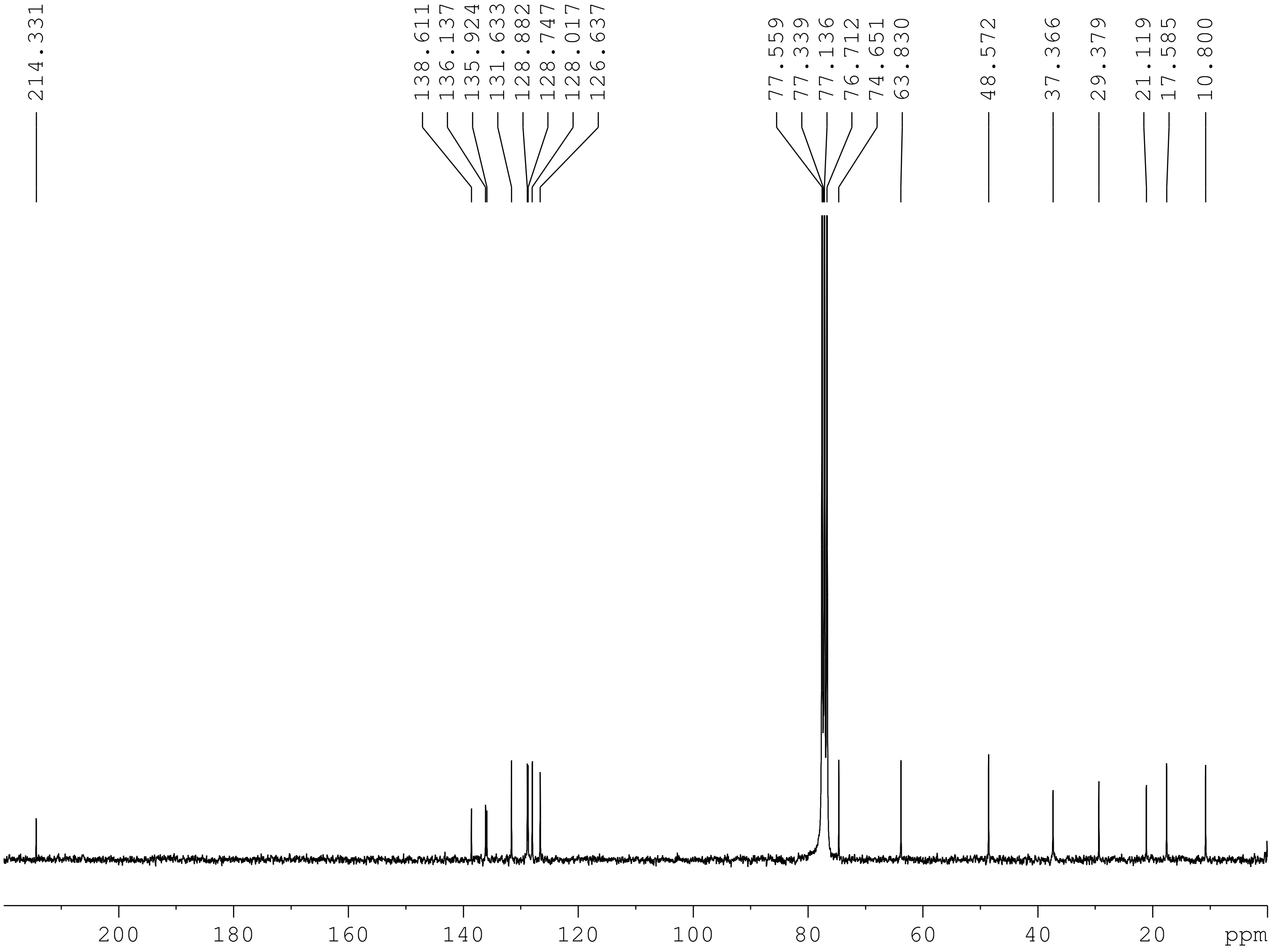

Current Data Parameters
NAME June08_BBO
EXPNO 892
PROCNO 1

F2 - Acquisition Parameters
Date_ 0
Time 17.19
INSTRUM spect
PROBHD 5 mm BBO BB-1H
PULPROG zgpg30
TD 16384
SOLVENT CDCl3
NS 12288
DS 8
SWH 18115.941 Hz
FIDRES 1.105709 Hz
AQ 0.4522484 sec
RG 1824.6
DW 27.600 usec
DE 6.00 usec
TE 0.0 K
D1 4.00000000 sec
d11 0.03000000 sec
DELTA 3.90000010 sec
MCREST 0.00000000 sec
MCWRK 0.01500000 sec

======== CHANNEL f1 ========
NUC1 13C
P1 8.00 usec
PL1 -2.00 dB
SFO1 75.4760376 MHz

======== CHANNEL f2 ========
CPDPRG2 waltz16
NUC2 1H
PCPD2 80.00 usec
PL2 1.00 dB
PL12 22.16 dB
PL13 22.00 dB
SFO2 300.1315007 MHz

F2 - Processing parameters
SI 32768
SF 75.4677397 MHz
WDW EM
SSB 0
LB 3.00 Hz
GB 0
PC 2.00
