# Supplementary material for: Fermentation, Isolation, Structure, and antidiabetic activity of NFAT-133 produced by Streptomyces strain PM0324667
Source: AMB Express. 2011 Nov 21;1:42. doi: 10.1186/2191-0855-1-42 (PMC3274447; doi:10.1186/2191-0855-1-42)
Supplement: Additional file 3 — 13C HSQC of the compound NFAT-133. The chromatogram represents the13C HSQC of the isolated compound NFAT-133 from the Streptomyces strain PM0324667. The sample ID for the compound was: 1111-41-1. [file 2191-0855-1-42-S3.PDF]

Piramal Life Sciences Limited

Sample ID:1111-41-1
Solvent : CDCl3
Method: 13C-HSQC
Instrument No: AS-I-09
Analyst: Rajendra
Date : 18.06.2008

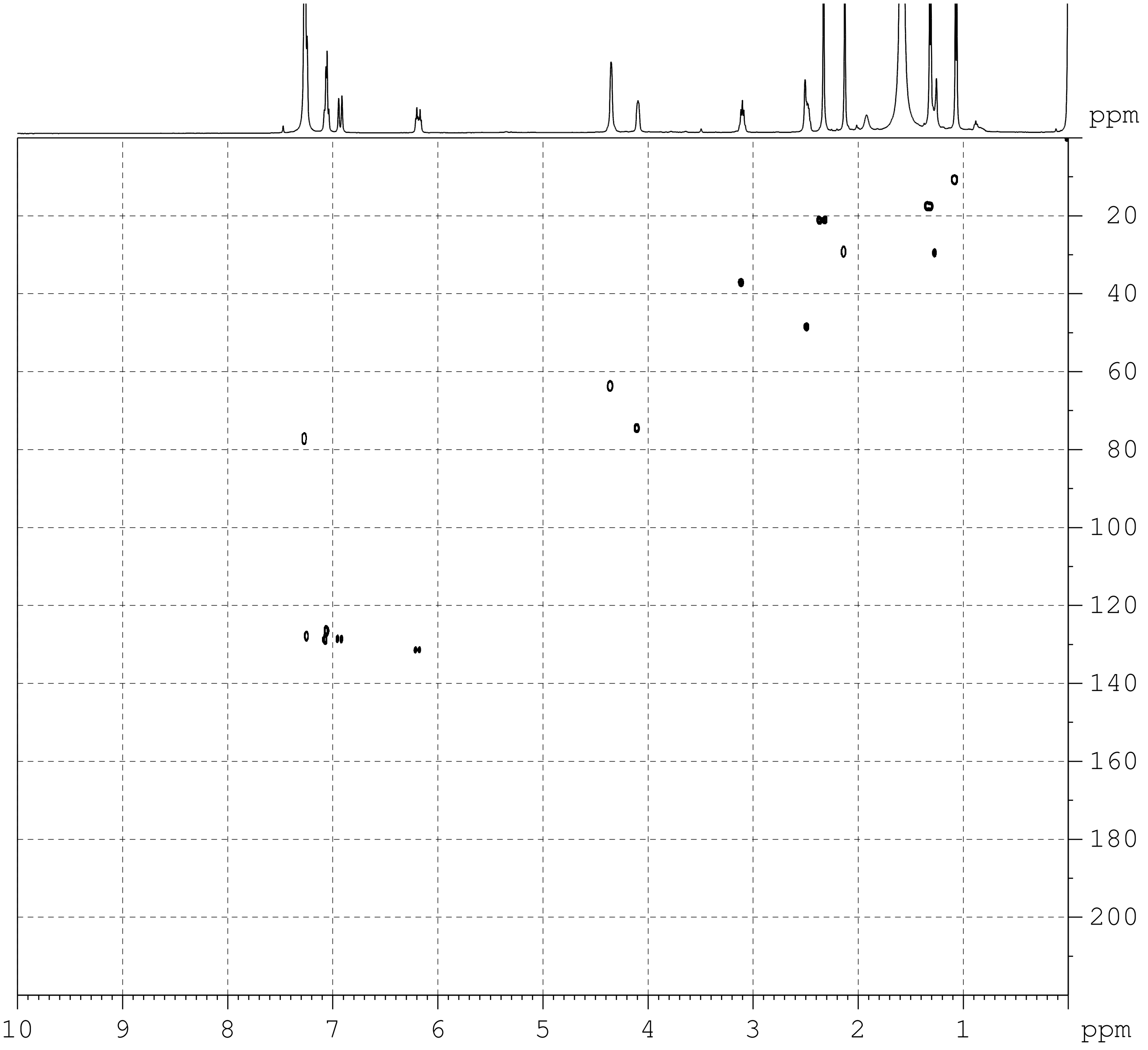

Current Data Parameters

NAME	Jun08_TXI	
EXPNO	127	
PROCNO	1	

F2 - Acquisition Parameters

Date_	20080619	
Time	13.33	
INSTRUM	spect	
PROBHD	5 mm TXI 1H/D-	
PULPROG	hsqcetgp	
TD	1024	
SOLVENT	CDCl3	
NS	64	
DS	16	
SWH	5000.000	Hz
FIDRES	4.882813	Hz
AQ	0.1025500	sec
RG	14600	
DW	100.000	usec
DE	6.00	usec
TE	295.1	K
CNST2	140.0000000	
d0	0.00000300	sec
D1	2.50000000	sec
d4	0.00178571	sec
d11	0.03000000	sec
d13	0.00000400	sec
D16	0.00020000	sec
DELTA	0.00122240	sec
DELTA1	0.00077771	sec
IN0	0.00001805	sec
ST1CNT	128	
ZGOPTNS		

======== CHANNEL f1 ========

NUC1	1H	
P1	8.20	usec
p2	16.40	usec
P28	0.00	usec
PL1	1.00	dB
SFO1	500.1825009	MHz

======== CHANNEL f2 ========

CPDPRG2	garp	
NUC2	13C	
P3	11.40	usec
p4	22.80	usec
PCPD2	70.00	usec
PL2	-4.00	dB
PL12	11.60	dB
SFO2	125.7828123	MHz

====== GRADIENT CHANNEL =====

GPNAM1	SINE.100	
GPNAM2	SINE.100	
GPZ1	80.00	%
GPZ2	20.10	%
P16	1000.00	usec

F1 - Acquisition parameters

ND0	2	
TD	128	
SFO1	125.7828	MHz
FIDRES	216.412735	Hz
SW	220.227	ppm
FnMODE	Echo-Antiecho	

F2 - Processing parameters

SI	1024	
SF	500.1800046	MHz
WDW	QSINE	
SSB	2	
LB	0.00	Hz
GB	0	
PC	1.40	

F1 - Processing parameters

SI	1024	
MC2	echo-antiecho	
SF	125.7703610	MHz
WDW	QSINE	
SSB	2	
LB	0.00	Hz
GB	0	
