# Supplementary material for: Fermentation, Isolation, Structure, and antidiabetic activity of NFAT-133 produced by Streptomyces strain PM0324667
Source: AMB Express. 2011 Nov 21;1:42. doi: 10.1186/2191-0855-1-42 (PMC3274447; doi:10.1186/2191-0855-1-42)
Supplement: Additional file 4 — DEPT-135 of the compound NFAT-133. The chromatogram represents the DEPT-135 of the isolated compound NFAT-133 from the Streptomyces strain PM0324667. The sample ID for the compound was: 1111-41-1. [file 2191-0855-1-42-S4.PDF]

Piramal Life Sciences Limited

Sample : 1111-41-1
Solvent : CDCl3
Spectrum : DEPT-135
Instrument: AS-I-10
Analyst : Jingal
Date : 26.06.2008

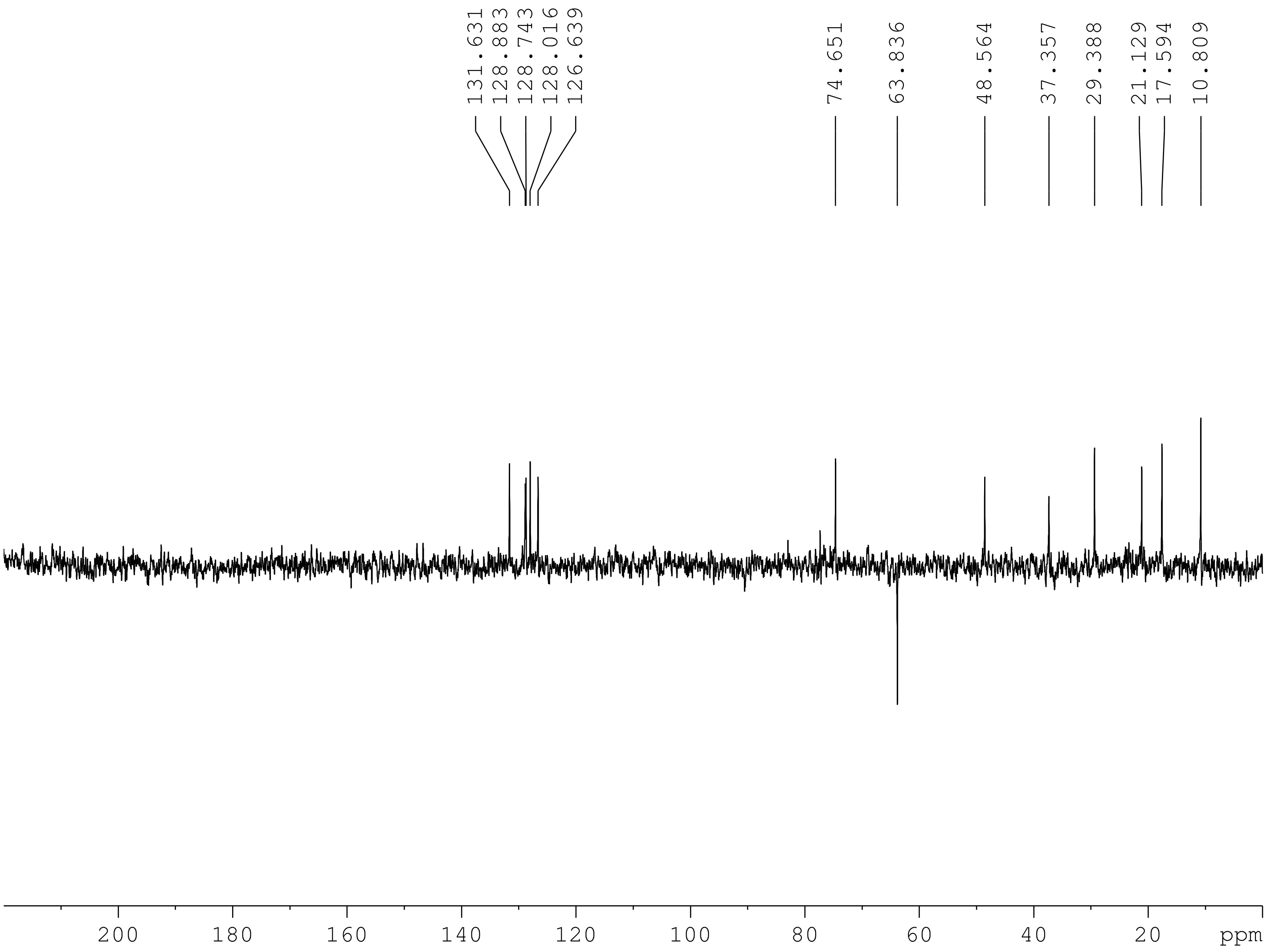

Current Data Parameters
NAME June08_BBO
EXPNO 893
PROCNO 1

F2 - Acquisition Parameters
Date_ 0
Time 10.53
INSTRUM spect
PROBHD 5 mm BBO BB-1H
PULPROG dept135
TD 65536
SOLVENT CDCl3
NS 10240
DS 8
SWH 18115.941 Hz
FIDRES 0.276427 Hz
AQ 1.8088436 sec
RG 23170.5
DW 27.600 usec
DE 6.00 usec
TE 0.0 K
CNST2 145.0000000
D1 4.00000000 sec
d2 0.00344828 sec
d12 0.00002000 sec
DELTA 0.00001019 sec
MCREST 0.00000000 sec
MCWRK 0.01500000 sec

======== CHANNEL f1 ========
NUC1 13C
P1 8.00 usec
p2 16.00 usec
PL1 -2.00 dB
SFO1 75.4752958 MHz

======== CHANNEL f2 ========
CPDPRG2 waltz16
NUC2 1H
P3 7.00 usec
p4 14.00 usec
PCPD2 80.00 usec
PL2 1.00 dB
PL12 22.16 dB
SFO2 300.1315007 MHz

F2 - Processing parameters
SI 32768
SF 75.4677400 MHz
WDW EM
SSB 0
LB 5.00 Hz
GB 0
PC 2.00
