# Supplementary material for: Fermentation, Isolation, Structure, and antidiabetic activity of NFAT-133 produced by Streptomyces strain PM0324667
Source: AMB Express. 2011 Nov 21;1:42. doi: 10.1186/2191-0855-1-42 (PMC3274447; doi:10.1186/2191-0855-1-42)
Supplement: Additional file 5 — 1H NMR of the compound NFAT-133. The chromatogram represents the1H NMR of the isolated compound NFAT-133 from the Streptomyces strain PM0324667. The sample ID for the compound was: 1111-41-1. [file 2191-0855-1-42-S5.PDF]

Piramal Life Sciences Limited

Sample ID:1111-41-1
Solvent : CDCl3
Method: 1H Spectrum
Instrument No: AS-I-09
Analyst: Rajendra
Date : 18.06.2008

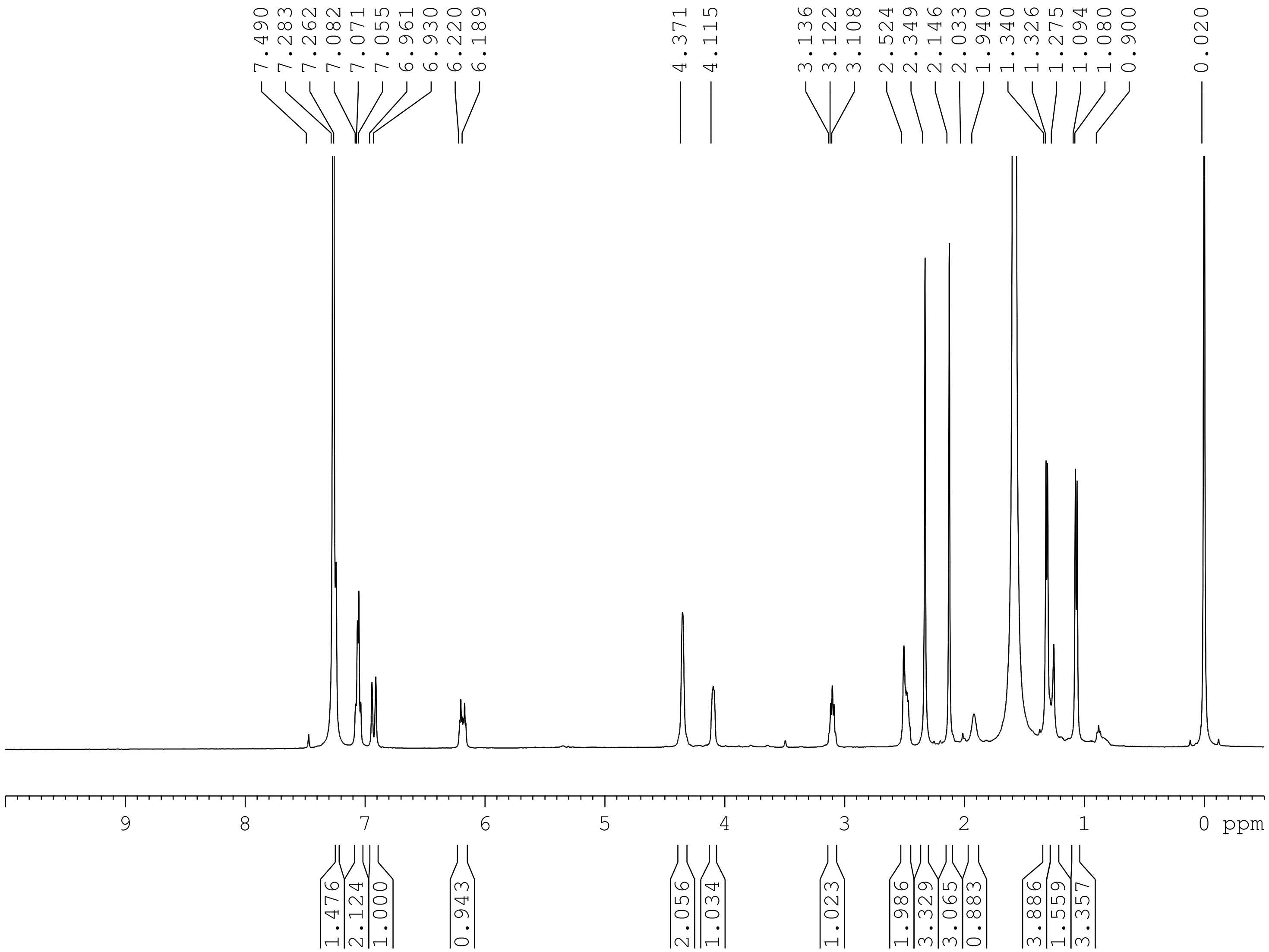

Current Data Parameters
NAME Jun08_TXI
EXPNO 125
PROCNO 1

F2 - Acquisition Parameters
Date_ 20080618
Time 17.17
INSTRUM spect
PROBHD 5 mm TXI 1H/D-
PULPROG zg30
TD 16384
SOLVENT CDCl3
NS 512
DS 0
SWH 10000.000 Hz
FIDRES 0.610352 Hz
AQ 0.8193000 sec
RG 575
DW 50.000 usec
DE 6.00 usec
TE 293.8 K
D1 2.00000000 sec
TD0 1

======== CHANNEL f1 ========
NUC1 1H
P1 8.20 usec
PL1 1.00 dB
SFO1 500.1842515 MHz

F2 - Processing parameters
SI 16384
SF 500.1800094 MHz
WDW EM
SSB 0
LB 3.00 Hz
GB 0
PC 1.00
